# From Etiology to Intervention: A Holistic Review of Bunion Pathophysiology and Care

**DOI:** 10.1155/2024/9910410

**Published:** 2024-10-11

**Authors:** Danielle Barnes, Paige Matijasich, Aidan Maxwell, David Yatsonsky, Audrey Ballard, Nabil Ebraheim, Osama Elattar

**Affiliations:** ^1^University of Toledo, College of Medicine and Life Sciences, Toledo, Ohio, USA; ^2^University of Toledo, Orthopedic Surgery Department, Toledo, Ohio, USA

## Abstract

In this review paper, we present the common etiology, presentation, diagnosis, and management of the following three common bunion formations: dorsal bunion, tailor's bunion, and hallux valgus (HV). Bunions are common pathologies that present to a variety of clinics, so it is important for providers to have a base understanding of these in order to provide the best care to patients. Many of these bunion formations have a variety of causes which allow providers to manage them before surgical intervention is required. The aim of this review paper is to bring attention and expanded insight on these common bunion presentations in order to minimize morbidity early on. The information provided in this review will allow both primary care and subspecialty physicians with the knowledge to accurately diagnose and optimally manage these bony deformities of the lower extremity.

## 1. Introduction

Bunions are a commonly seen pathology not only by orthopedic surgeons and podiatrists but also in primary care and family medicine practices. They are a very common foot deformity, affecting 23% of 18–65-year olds and being more common with older age [1]. Although the term “bunion” is commonly associated with hallux valgus (HV) deformity, it is a lay term used to describe an inflamed bony prominence and its overlying bursa [2, 3]. This article focuses on the etiology, presentation, diagnosis, and management of the following three types of bunions: dorsal bunion, tailor's bunion, and HV. By describing these bunions and underlying etiologies, physicians and other healthcare providers will be provided a well-rounded resource on how to manage and treat the most prevalent bunion pathologies.

## 2. Dorsal Bunion

### 2.1. Etiology

Dorsal bunion, also termed hallux flexus, is a deformity where the first metatarsal head is in a dorsiflexed position, the metatarsophalangeal (MTP) joint is plantar flexed, and its interphalangeal (IP) joint is extended [2, 4]. It is primarily due to a muscle imbalance that leads to dorsal subluxation and prominence of the first metatarsal head [2, 4]. The muscles involved in this imbalance include the peroneus longus muscle, flexor hallucis brevis muscle, tibialis anterior muscle, and gastrocnemius soleus muscle. One imbalance is between the tibialis anterior and the peroneus longus. The tibialis anterior muscle acts as an antagonist to the peroneus longus, which acts to stabilize the first metatarsal head to the ground while standing and at the push-off phase during walking [2]. The overactivity of the tibialis anterior muscle or the underactivity of the peroneus longus tendon leads to an imbalance between the flexors of the hallux which are strong and the extensors of the hallux which are weak, leading to unopposed dorsiflexion of the first metatarsal [2, 5, 6]. A study by McKay discussed how gastrocnemius weakness can lead to compensation by the strong secondary plantar flexors, such as the flexor hallucis longus and brevis, thus leading to plantar flexion at the MTP join and exacerbating dorsal bunion formation [6, 7]. Although there are many etiologies described throughout literature, almost all discuss muscular imbalances leading to altered foot mechanics as a major contributing factor.

The dorsal bunion deformity mainly occurs as a sequela to congenital clubfoot and paralytic deformities of the foot such as Charcot–Marie–Tooth syndrome, poliomyelitis, and cerebral palsy [2–4, 8–10]. Therefore, it is more commonly seen in children [2–4, 8–10]. A dorsal bunion can be a residual deformity of the clubfoot pathology or can develop as a complication of surgical correction of clubfoot deformity [2–4, 8]. One study by Kuo summarized that the major factors leading to dorsal bunion after clubfoot surgery include weakness of the gastrocnemius soleus complex, a strong overpowering flexor hallucis longus, forefoot supination with a strong anterior tibial tendon, and weakness of the peroneal longus tendon [5, 9].

Hallux rigidus, meaning “stiff big toe,” also commonly leads to the formation of a dorsal bunion [2, 4]. Hallux rigidus is a type of degenerative osteoarthritis of the first MTP that causes rubbing of the articular surfaces, leading to the formation of dorsal osteophytes. These osteophytes form a bump on top of the joint [4]. The pathophysiology of hallux rigidus is not well known but there are some studies that suggest iatrogenic and traumatic injuries as a cause [4]. One study suggests that hallux rigidus is caused by the limitation to normal movements of flexion and extension (sagittal plane) leading to joint degeneration [11]. These altered mechanics overtime can cause displacement of the weight-bearing area under the first metatarsal head under the IP joint which contributes to the development of the dorsal bunion [2].

Some additional less common etiologies include global forefoot supination, poliomyelitis, disc herniation, and compartmental syndrome [2–4, 8].

### 2.2. Clinical Presentation

Patients often present with complaints of deformity (dorsal prominence and callosities) and pain (dorsal 1^st^ metatarsal head and metatarsalgia). They may have a history of cerebral palsy, poliomyelitis, compartment syndrome, disc herniation, Charcot–Marie–Tooth syndrome, or corrected clubfoot deformity [2–4, 8].

In terms of hallux rigidus, the patient complains of pain that is worse with activities. There may also be complaints of joint stiffness, joint locking, or an altered (everted) gait. The pain may improve with the removal of shoes [2–4, 8]. The motion of the joint is restricted, especially in dorsiflexion [2–4, 8, 11]. In the seated position, the first MTP is often tender with palpable dorsal osteophytes [2–4, 8]. In some cases, there may be dorsal skin compromise over the osteophyte with thickening of the dorsal skin, redness or blistering, or ulceration [11].

Patients typically present to their primary care doctor once they start getting symptoms such as pain, difficulties with footwear, gait disruption, and skin compromise [10, 12].

### 2.3. Diagnosis

**Figure 1:**
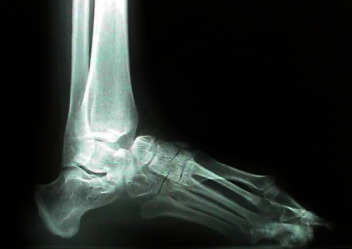
X-ray of the left foot, showing the horizontal alignment of the first metatarsal, flexion of the first MTP and an extension of the IPP joint of the hallux [2].

Diagnosis is established by thorough history taking, physical examination, and radiographic evaluation. Diagnosis is typically made clinically. Clinical features almost always present include the first metatarsal head in a dorsiflexed position, the MTP joint plantar-flexed, and the IP joint extended [2, 4]. Physical examinations may also demonstrate a dorsal bony prominence at the first metatarsophalangeal joint (MTPJ). The physical changes can also be accompanied by metatarsalgia, commonly under the central metatarsal heads but can also be located laterally [2]. There may be swelling and erythema accompanying the mechanical and physical changes. Blood work can be used to rule out other causes of inflammation in the joint, such as gout, rheumatoid arthritis, or septic arthritis. Radiographic evaluation includes weight-bearing films, anteroposterior, lateral, and oblique. A study by Albert described hallux flexus imaging which showed horizontal alignment of the first metatarsal, flexion of the 1^st^ MTPJ and an extension of the interphalangeal joint (IPP) joint of the hallux (Figure 1) [2]. With hallux rigidus, the lateral radiograph often reveals a large dorsal osteophyte at the head of the metatarsal [4, 11]. Other findings indicative of hallux rigidus may include joint space narrowing, subchondral cyst formation, and sclerosis [11]. In the initial stages of the disease, radiographs may look normal or may only show findings confined to the dorsal aspect of the joint such as soft tissue fullness over the metatarsal head [11]. As the disease progresses, radiographs will show complete narrowing of the joint space on the AP view and proximal phalangeal and distal metatarsal head osteophytes on the lateral radiograph view [11]. The presence of hallux rigidus should prompt further evaluation for hallux flexus.

### 2.4. Management

#### 2.4.1. Nonoperative

A nonoperative approach can be effective if the deformity is mild, flexible, and not causing severe discomfort to the patient. For patients experiencing hallux rigidus, a nonoperative approach should be tried prior to surgical treatments, ideally preventing a dorsal bunion. These treatments include oral nonsteroidal anti-inflammatory drugs (NSAIDS), intra-articular injections, shoe modification, activity modification, and physical therapy. In the early stages of hallux rigidus and hallux flexus, the first approach usually begins with shoe modifications and foot orthoses, designed to limit irritation from the dorsal osteophytes, reducing motion and the mechanical stresses on the joint [11, 13–15]. NSAIDs have been demonstrated not to be effective alone and work best when combined with one or more of the other treatment options listed above [13–15]. Shoe modification and orthotics reduce pain by modifying the biomechanics of the first MTPJ [16]. Rocker bottom shoes, for example, can help reduce painful dorsiflexion by allowing the patient to transition from heel strike to toe-off in the gait cycle without requiring the foot or shoe to bend [11]. Shoes with high toe boxes can prevent direct contact between the dorsal osteophytes and the shoe and therefore relieve the pressure from the 1^st^ MTPJ [3]. Physical therapies may also be used and involve joint mobilization, manipulation, and improving range of motion [13]. However, more needs to be done with exploring these alternative managements, as evidence is still lacking [17].

#### 2.4.2. Operative

Operative management is typically explored for reasons including aesthetics, pain, walking instability, or ineffective conservative management [10]. There is no standardized surgical correction method that applies to all dorsal bunions, and the surgery is often tailored to each individual case and dictated by the etiology of the deformity. Various surgical treatments have been discussed including plantarflexion osteotomy of the first ray (first metatarsal and first cuneiform bones) plus arthrodesis of the metatarsal-cuneiform and cuneiform-navicular joints, excision of the proximal portion of the proximal phalanx, various tendon transfers about the hallux, and arthrodesis of the first MTP joint [7, 10, 12]. In summary, surgical corrections can be as simple as performing a tendon or soft tissue release and cheilectomy or a more complex procedure that involves tendon transfer, osteotomy, or 1^st^ MTPJ fusion.

### 2.5. Tailor's Bunion

#### 2.5.1. Etiology

Tailor's bunion, synonymously known as the bunionette deformity, is a bony prominence of the lateral fifth metatarsal head [18–20]. The term “tailor's bunion” is historically derived from the increased frequency in which the deformity was seen in tailors who frequently place pressure on the lateral aspect of the foot while sitting with their legs crossed [18, 21, 22]. There is a female and athlete predominance in adults and adolescents [23–25]. There are various lifestyle factors that are known to contribute to these deformities, such as wearing narrow or pointed shoes which crowd the toes. Inflammatory diseases such as rheumatoid arthritis and lupus have been shown to be involved in the formation of these deformities as well [24]. The causes of the deformity can be divided into anatomical and biomechanical causes [23–25]. Anatomical causes include increased pressure over the lateral aspect of the fifth metatarsal due to a tight shoe or foot positioning, a prominent lateral condyle with excessive friction, and hypertrophy of the soft tissues over the lateral aspect of the metatarsal head [21, 24]. Biomechanical causes include lateral angulation (bowing) of the fifth metatarsal, increased angular deviation between the fourth and fifth metatarsal shafts (intermetatarsal angle [IMA]), and excessive pronation due to hypermobility [21, 24]. The bunionette deformity is classified based on the findings observed on weight bearing dorsoplantar radiographs [21, 25]:

  Type I is an enlargement of the lateral aspect of the fifth metatarsal head. This can be due to exostosis, a prominent lateral condyle or a round dumbbell-shaped metatarsal head [26]. With excessive pronation of the foot, the lateral plantar tubercle of the fifth metatarsal head rotates laterally to create the radiographic impression of an enlarged fifth metatarsal head [21, 25].
  Type II is secondary to abnormal lateral bowing of the distal fifth metatarsal with a normal 4-5 IMA. There is usually no associated hypertrophy of the fifth metatarsal head [21, 25].
  Type III is the most observed and is characterized by an increase 4-5 IMA with divergence of the fourth and fifth metatarsals [21, 25].
  Type IV is quite uncommon and consists of a combination of deformities including two or more from the list above. This is most seen in patients with rheumatoid arthritis and frequently resistant to conservative treatment [21].

#### 2.5.2. Clinical Presentation

The deformity is often asymptomatic and an incidental finding [23]. When the tissue overlying the deformity becomes calloused or inflamed, patients often seek treatment [23]. The patient presents primarily with a subjective complaint of pain over the lateral, plantar, or dorsal aspect of the fifth metatarsal head [22]. This pain is particularly present with the wearing of constrictive shoes and ambulation [21]. With the continuous pressure and chronic irritation over the exostosis, a secondary hyperkeratotic lesion can occur, most commonly on the lateral aspect [22, 27]. A bursa may also develop and worsen due to repeated activities that lead to thickening or inflammation of the bursa [27]. Erythema and edema of the fifth toe is commonly present [21, 23, 27].

#### 2.5.3. Diagnosis

Diagnosis is based on patient history, physical examination, and radiographic evaluation. Physical examination may demonstrate pes planus upon weight bearing [19]. There may also be the coexistence of HV in those with an increase in forefoot width as the deformities share common risk factors [19, 23, 27]. The patient should be evaluated for associated swelling, erythema, keratosis, and ulceration of the lateral aspect of the fifth metatarsal [19, 23]. Through physical exam findings and history taking, other causes of the presenting symptoms should be ruled out such as gout, septic arthritis, turf toe, rheumatoid arthritis, and Morton's neuroma. The location of callous should be noted, whether plantar, dorsal, or lateral, as it may represent pattern of wear and guide surgical approach [19, 23].

**Figure 2:**
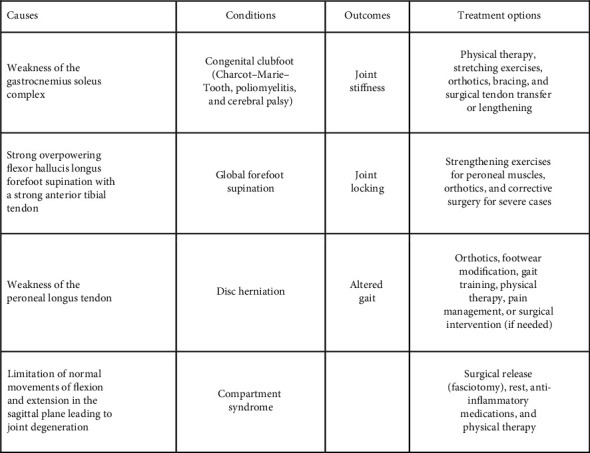
Table summarizing the causes, conditions, outcomes and treatment options of dorsal bunions.

In terms of radiographic evaluation, anteroposterior and lateral, plain foot, weight-bearing radiographs are the standard [21, 22]. Oblique radiographs can be helpful in evaluating the fifth metatarsal head, lateral tubercle, metatarsal deviation, and lateral soft tissue prominence [21]. Nonbiomechanical causes of the lateral prominence, such as fracture, and tumor should be ruled out [22]. The most common method of evaluating the deformity is measuring the 4-5 IMA and the fifth metatarsopmhalangeal or lateral deviation angle (Figure 2) [21]. The 4-5 IMA is formed by two lines that bisect the fourth and fifth metatarsals and found to be abnormal with an angle > 8 degrees based on Coughlin classification [22, 23]. The fifth MTP angle is measured by the degree of divergence of the fifth toe from the long axis of the metatarsal shaft and found to be abnormal with an angle > 14 degrees [22, 24].

### 2.6. Management

#### 2.6.1. Nonoperative

No evidence-based guidelines exist for the conservative management of the bunionette deformity; however, there are several recommendations agreed upon [24]. Nonsurgical management should be considered initially for all bunionette deformities [24, 28]. Conservative management is often successful in treating symptomatic bunionettes [24]. It is recommended that the patient option for shoes with wide toe box as opposed to narrow or high heeled shoes as constricting footwear is a significant cause of symptoms due to the increased pressure on the prominent fifth metatarsal [19]. If acute pain is present due to an inflamed bursa, treatment with NSAIDS and anti-inflammatory medications, as well as steroidal injections, have been found to be helpful in relieving pain [21]. If a hyperkeratotic lesion is present, this can be padded, or debridement of the lesion can offer relief of pressure and pain [10]. When pain over the fifth metatarsal head is caused by abnormal foot mechanics, the use of orthotics can alleviate symptoms and decrease pronation of the foot.

#### 2.6.2. Operative

Surgery is indicated when nonoperative management can no longer control symptoms or if the patient is highly active [19, 21, 23, 27]. Surgical intervention aims to decrease the width of the foot and the prominence of the bunionette [19]. In addition, the preservation of function of the fifth MTPJ may prevent complications such as recurrence, subluxation, dislocation, or the development of a transfer lesion [19, 21–24, 27]. The surgeon may option for a simple lateral eminence resection or an osteotomy to correct the deformity. The type of procedure used to correct the deformity is dictated by the type and severity of the Bunionette deformity [19, 21–24, 27].

### 2.7. HV

#### 2.7.1. Etiology

HV is the most common type of bunion, with reported incidence of 3.5% in adolescence, 23% of the population aged 18–65 and up to 36% over 65 years [29–31]. By definition, HV is a deformity with lateral (valgus) deviation of the great toe and medial (varus) deviation of the first metatarsal [29]. Although the etiologies of HV can be complex, many factors have been associated with HV including genetic predisposition, structural factors, sex, age, BMI, foot pain, pes planus and footwear [32]. One commonly discussed cause for deformity is ill-fitting shoes. Women are more commonly affected than men, which is thought to be due to the fact that they wear narrow, high-heeled shoes and often have more flexible soft tissues [32, 33]. HV deformity is also associated with rheumatoid arthritis, especially in the chronic stages, affecting 60%–90% of chronic rheumatoid arthritis patients [29, 34]. Other associated arthritic conditions include gouty arthritis, psoriatic arthritis [29, 35]. HV deformity can also commonly be seen in connective tissue disorders such as Marfan syndrome and Ehlers–Danlos syndrome, as well as in Down syndrome [29, 35]. Anatomically, HV can also be associated with short first metatarsal, dorsiflexed first metatarsal, flexible, or rigid forefoot varus, rigid or flexible pes planovalgus or gastrocnemius equinus, abnormal foot mechanics, and joint hypermobility [29, 35]. Muscle imbalances in the foot can also predispose patients to getting the HV deformity. These muscle imbalances can be caused by conditions such as a stroke, cerebral palsy, or myelomeningocele [35].

The muscle imbalance leading to HV likely exists between the extrinsic and intrinsic muscles of the foot with involvement of the ligaments [35]. The muscles involved include the peroneus longus, abductor hallucis, and adductor hallucis. The peroneus longus pulls the first metatarsal laterally while the abductor hallucis muscle creates tension by pulling the first metatarsal medially. This maintains the alignment of the first metatarsal [35]. Collateral ligaments prevent movement along the transverse plane at the first MTP joint [35, 36]. In the case of HV, an imbalance occurs resulting in more tension pulling the first metatarsal medially paired with the likely underactivity of musculus abductor hallucis and overactivity of the musculus adductor hallucis, resulting in the HV deformity [37].

#### 2.7.2. Presentation

HV bunions present as deformities of the great toe [29–31]. They are progressive problems, which may cause pain, soreness, burning sensation, and redness [29–31]. Most commonly HV presents in aging females [29]. HV bunions are most symptomatic when wearing shoes with a tight shoe box such as high heels [29]. This condition is frequently associated with hammer toe deformity of the lesser toes, which by definition is an extension deformity of the DIP joint, with flexion at the PIP joint and slight extension of the lesser MTP joint [29–31]. HV is also associated with callus formation, sesamoid arthritis in advanced cases. Common associated deformities include pes planus, lesser toe deformities, and various midfoot/hindfoot conditions [31].

**Figure 3:**
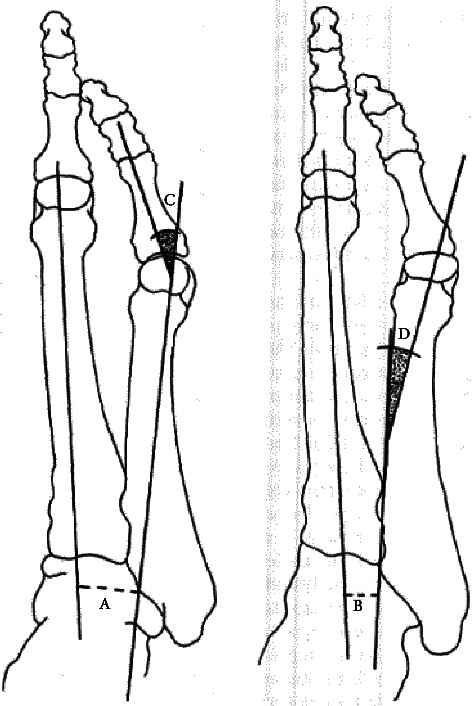
A. 4-5 metatarsal angle; B. lateral deviation angle [26].

Juvenile hallux valgus (JHV) and adolescent hallux valgus (AHV) are variations of the HV pathology [38]. JHV and AHV may be distinguished from the adult variation because they often present bilaterally with familial predilection [38]. These are typically less painful. In these populations, congenital varus deviation of the first metatarsal (metatarsus primus varus) with subsequent increased IMA between the 1^st^ and 2^nd^ metatarsal shafts is typically present (Figure 3) [31]. The dorsal metatarsal angle (DMAA) is also increased in most JHV and AHV (Figure 3) [38].

#### 2.7.3. Diagnosis

Diagnosis can be made through thorough history, meticulous physical exams, and plain radiographs [29–31, 40]. Establishing a diagnosis can typically be done through physical exam only, without laboratory testing or radiological studies. However, imaging can help determine the extent of the deformity and give guidance in treatment decisions [35].

A thorough biomechanical exam will also be useful in determining the cause and treatment of the deformity [35, 41]. Evaluation of the deformity and probable causes are usually divided into weight-bearing and nonweight-bearing physical examination [35]. HV deformity severity tends to be more obvious with weight bearing [35]. On physical examination, the hallux will be deviated laterally and pronated (rotated) due to deviation of the proximal phalanx promoting varus position of the 1^st^ metatarsal [29]. It is often accompanied by callous formation, sesamoid pain, and arthritis [29]. Further physical exam should focus on the first MTP range of motion and 1^st^ tarsometatarsal joint mobility [29–31]. As noted by Kuhn and Alvi, common items to evaluate should include forefoot/rearfoot varus or valgus, subtalar joint stiffness, midtarsal joint stiffness, resting calcaneal stance position, tibial torsion, and neutral calcaneal stance position [35].

**Figure 4:**
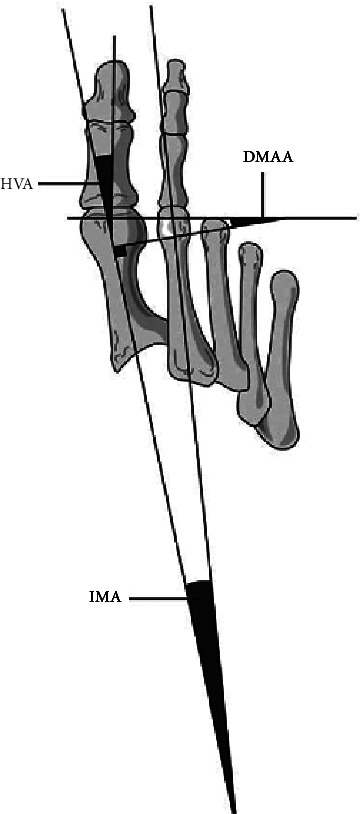
Radiologic assessment and presentation of hallux valgus angles. Demonstrating the various angles: intermetatarsal angle (IMA), dorsal metatarsal angle (DMAA), and first metatarsophalangeal angle, also known as hallux valgus angle (HVA) [39].

Imaging can be used to determine the severity of the deformity and give insight into the treatment options. X-rays should include anteroposterior and lateral weight-bearing radiographs of the entire foot and are necessary for adequate assessment of the deformity [29, 31]. Oblique and sesamoid views can also be beneficial [31]. Radiographic findings include displacement of the sesamoids laterally, changes in joint congruency and possibly degenerative changes [30, 31, 40]. The radiographic findings are often used to classify the HV deformity based on varying angle measurements. One pertinent measurement includes HVA (the angle subtended by the long axes of the first MT and proximal phalanx) which identifies MTP deformities [31]. A normal HVA is less than 15 degrees [29–31, 42]. As shown in Figure 4, IMA is another useful radiographic benchmark and is measured between the long axes of the first and second metatarsal shafts, with a normal angle less than 9 degrees [39, 42]. A severe HV is characterized by an IMA greater than or equal to 20 degrees [29–31, 35]. Additional measurements include DMAA, which is the angle between the first MT axis and the perpendicular to the base of the distal articular cap [42]. DMAA can identify MTP joint congruity vs. incongruity, with a normal less than 10 degrees. Hallux valgus interphlangeus (HVI) is another radiographic measurement useful for identification and classification of HV deformity [43]. HVI is the angle between the long axis of distal and proximal phalanges, with a normal angle less than 10 degrees [43]. These measurements can help guide referrals and definitive management by aiding in the classification of the deformity into normal, mild, moderate, and severe. Utilization of advanced imaging methods such as ultrasound and magnetic resonance imaging (MRI) has become an emerging tool in diagnosis due to their ability to confirm clinical suspicion and play a key role in management. Although MRI provides reliable soft tissue resolution, ultrasound is the firstline tool used in most patients to evaluate musculoskeletal disorders because of its high resolution for soft tissue, low cost, availability, and excellent patient tolerance. Ultrasound also offers the ability to provide a dynamic assessment, allowing physicians to localize and characterize pathologies of the foot quickly and accurately [44].

### 2.8. Management

#### 2.8.1. Nonoperative

There are several methods of treatment for HV, especially in the early stages. Although there is no definitive research that shows conservative treatment if effective, nonoperative treatment is still typically tried first [35]. The nonoperative management options mostly focus on symptomatic treatment, and it is important to educate the patients that these modalities do not correct the deformity [30, 35, 40]. Firstline conservative measures include things such as shoe modification, avoiding tight shoe boxes and high heels, wearing orthoses to improve alignment and support, analgesics, icing to reduce inflammation, stretching, and wearing median bunion pads to reduce inflammation [35, 42]. Another more recent treatment being explored is muscle strengthening. A study by Glasoe addresses the actions of 5 muscles that have been identified as having the ability to counter the HV process: the abductor hallucis, adductor hallucis, flexor hallucis brevis (the intrinsic muscles), tibialis posterior, and fibularis longus (the extrinsic muscles) [45]. As with the other conservative measures, muscle strengthening would not correct the deformity, but it would reduce the pain and ideally address the gait impairments. Many of these conservative measures are done to help control and reduce pain, allowing patients with HV to live more comfortably [35, 45]. If the pain is not well controlled, the conservative measurements are considered to have failed, and the next steps are likely discussions regarding operative management.

#### 2.8.2. Operative

Surgical intervention is indicated if symptoms are not controlled with conservative therapy [40]. The goals of surgery are to correct deformity, improve pain, and maintain motion of the 1^st^ MTP joint [29, 33, 46]. Contrary to the false belief, cosmesis is not the goal of surgery [33]. The technique/approach for surgical correction will vary based on the patient's deformity, age, health, and surgeon preferences [29, 33, 46, 47]. Details of each procedure can be found in relevant literature, but the standard operative protocol varies based on degree of deformity as a general rule. For example, distal osteotomies are indicated in mild disease (IMA < 13) and proximal or combined osteotomies are indicated in more moderate disease (IMA > 13). Other surgical techniques include 1st TMT arthrodesis for arthritis at TMT joint or instability and fusion procedure for severe deformity/spasticity/arthritis. MTP resection arthroplasty is only indicated in elderly patients with low functional demands and soft tissue procedure is almost never indicated [29, 33, 35, 46]. It is important to note that although surgery is the only treatment to correct this deformity, there is a 15% recurrence rate [45]. As mentioned, the surgical technique utilized varies greatly depending on the degree and deformity; however, the standard of care following osteotomies are remaining nonweight bearing for two weeks following the procedure, followed by partial progressive weight bearing for the next four weeks. This differs from patients who undergo fusion procedures, as they are nonweight bearing for six to eight weeks following the surgery [29, 33, 46]. Regarding juvenile and adolescent HV deformity, surgical corrections are preferably performed once patients reach skeletal maturity [38]. Proximal metatarsal osteotomies cannot be performed if physis are open [38]. Medial cuneiform osteotomies, however, are possible prior to physis closure [38]. When possible, it is ideal to pursue conservative measures until skeletal maturity has been reached due to the reported elevated risk of recurrence with attempted surgical correction with open physes [38].

## 3. Conclusion

The primary care provider plays a key role in addressing the concerns of a patient presenting with a bunion deformity. As conservative management is often the first step for treatment, primary care physicians can provide symptomatic relief and appropriate guidance for further management. It is also important to understand the indications for possible operative treatment, as primary care physicians are often the first point of contact for patients who present with a bunion deformity. Knowledge of these types of bunion deformities can expedite treatment and recovery and decrease patient morbidity. A better understanding of the etiology and presentation of these bunion deformities can assist both primary care and subspecialist providers with a roadmap to successfully manage and diagnose patients. Continued research within this field of bony deformities is necessary to better understand genetic predispositions and their impacts on both operative and nonoperative management options for patients.

## Data Availability

The data that support the findings of this study are available in the public resources PubMed and Google Scholar. Literature searches were completed on google scholar to collect information to create this review.
